# The Nursing Home Severity Index and Application to Pressure Injury Risk: Measure Development and Validation Study

**DOI:** 10.2196/43130

**Published:** 2023-02-09

**Authors:** Tracey L Yap, Susan D Horn, Phoebe D Sharkey, Katie R Brooks, Susan Kennerly

**Affiliations:** 1 School of Nursing Duke University Durham, NC United States; 2 School of Medicine University of Utah Salt Lake City, UT United States; 3 Sellinger School of Business Loyola University Maryland Baltimore, MD United States; 4 College of Nursing East Carolina University Greenville, NC United States

**Keywords:** geriatrics, nursing homes, pressure ulcer, propensity scores, severity of illness index, development, validation, clinical, treatment, pressure injury, injury, risk, prevention

## Abstract

**Background:**

An assessment tool is needed to measure the clinical severity of nursing home residents to improve the prediction of outcomes and provide guidance in treatment planning.

**Objective:**

This study aims to describe the development of the Nursing Home Severity Index, a clinical severity measure targeted for nursing home residents with the potential to be individually tailored to different outcomes, such as pressure injury.

**Methods:**

A retrospective nonexperimental design was used to develop and validate the Nursing Home Severity Index using secondary data from 9 nursing homes participating in the 12-month preintervention period of the Turn Everyone and Move for Ulcer Prevention (TEAM-UP) pragmatic clinical trial. Expert opinion and clinical literature were used to identify indicators, which were grouped into severity dimensions. Index performance and validation to predict risk of pressure injury were accomplished using secondary data from nursing home electronic health records, Minimum Data Sets, and Risk Management Systems. Logistic regression models including a resident’s Worst-Braden score with/without severity dimensions generated propensity scores. Goodness of fit for overall models was assessed using C statistic; the significance of improvement of fit after adding severity components to the model was determined using the likelihood ratio chi-square test. The significance of each component was assessed with odds ratios. Validation based on randomly selected 65% training and 35% validation data sets was used to confirm the reliability of the severity measure. Finally, the discriminating ability of models was evaluated using propensity stratification to evaluate which model best discriminated between residents with/without pressure injury.

**Results:**

Data from 1015 residents without pressure injuries on admission were used for the Nursing Home Severity Index–Pressure Injury and included laboratory, weights/vitals/pain, underweight, and locomotion severity dimensions. Logistic regression C statistic measuring predictive accuracy increased by 19.3% (from 0.627 to 0.748; *P*<.001) when adding four severity dimensions to Worst-Braden scores. Significantly higher odds of developing pressure injuries were associated with increasing dimension scores. The use of the three highest propensity deciles predicting the greatest risk of pressure injury improved predictive accuracy by detecting 21 more residents who developed pressure injury (n=58, 65.2% vs n=37, 42.0%) when both severity dimensions and Worst-Braden score were included in prediction modeling.

**Conclusions:**

The clinical Nursing Home Severity Index–Pressure Injury was successfully developed and tested using the outcome of pressure injury. Overall predictive capacity was enhanced when using severity dimensions in combination with Worst-Braden scores. This index has the potential to significantly impact the quality of care decisions aimed at improving individual pressure injury prevention plans.

**Trial Registration:**

ClinicalTrials.gov NCT02996331; http://clinicaltrials.gov/ct2/show/NCT02996331

## Introduction

The aging of the population has resulted in over 1.3 million residents living in nursing home facilities in the United States [1]. Improving the quality of care and containing overall costs will require substantial research efforts to find solutions for how to provide optimal levels of care to these residents. More specifically, an area requiring quality of care improvement for this nursing home population is understanding how to prevent pressure injuries (PrIs), given our inability to control the associated pain, infection, and the potential for death once the injury develops. Current prevention approaches have not been as effective as needed; in fact, just being a nursing home resident increases one’s risk of developing a PrI [2]. Nursing home prevention care is guided by the international PrI prevention guidelines [3] that advocate for risk assessment. The Braden Scale for Predicting Pressure Sore Risk (hereafter Braden score) [4] is a commonly used assessment tool to quantify PrI risk represented by a total score and risk categories (low, mild, moderate, high). Many practitioners focus their prevention efforts on residents considered at moderate or high risk. However, PrI incidence remains high among all residents regardless of the Braden score–assessed risk category [5]. The high prevalence of residents who are severely ill makes determining overall PrI risk challenging. Resident attributes beyond the Braden score may add insights to help discriminate those who are at risk of a PrI developing.

Clinical severity, the extent of physiologic decompensation, reflects the overall complexity of a resident’s health status. The severity of illness measures initially were developed in the 1980s using supervised techniques that predict a specific target value, applying statistical methods with historical data. These measures helped to explain why patient mortality, cost, or length of stay differed among hospitals. Their ability to accurately predict a variety of outcomes, however, was limited given that patient attributes were in part defined by specified treatments and created using regression analyses to predict one outcome [6-9].

The Comprehensive Severity Index (CSI) [10,11], which was also developed in the mid-1980s for the evaluation of overall clinical severity levels, applied a substantively different unsupervised method based on judgments of disease-specific medical experts, literature, and clinical textbooks rather than statistical methods, specific outcomes, and use of historical data. This objective measure of clinical severity used physiological, functional, and psychosocial data, including demographics and over 2200 diagnosis-specific signs, symptoms, and physical findings (no treatments). The methodology and CSI are well established and have been validated extensively for over 30 years in patients with many different clinical conditions [10-18].

The development of a clinical severity measure targeted for nursing home residents requires the construction of a new measure with the potential to be individually tailored to different outcomes, such as a PrI. The CSI and other existing clinical severity measures are not appropriate for use with nursing home residents who have large variations in their length of stay, the time windows for data collection, and documentation frequency. This paper reports on the creation of a new clinical severity measure, Nursing Home Severity Index (NHSI), tailored for PrI risk prediction for nursing home residents, and validation using propensity modeling to predict PrI development and explore the measure’s predictive accuracy beyond the Braden score.

## Methods

### Overview

Development of the NHSI and the selected attributes associated with PrI risk (NHSI-PrI) required initial variable selection, scoring, validation, and propensity modeling to account for independent and confounding variables that affect PrI development and exploration of the measure’s predictive accuracy and validity. A retrospective nonexperimental design was used to examine a broad range of resident attributes to develop the NHSI-PrI. Current study data were based on a 12-month longitudinal data set from each study nursing home to account for seasonal differences.

### Study Setting and Population Sample

Residents from 9 skilled nursing homes participating in a 12-month preintervention period (hereafter study period) of the Turn Everyone And Move for Ulcer Prevention (TEAM-UP) embedded pragmatic cluster randomized trial (R01NR016001; ClinicalTrials.gov NCT02996331) [5,19] were involved in this aspect of the study. All 9 participating Medicare and Medicaid–certified skilled nursing homes with ≥100 operating beds were in the same long-term care company and used the same electronic health record (EHR) systems with comprehensive resident clinical information. The population sample included all nursing home residents aged ≥18 years without an existing PrI on study period entry and without regard to diagnoses or demographic attributes.

### Ethics Approval

Duke University Institutional Review Board (Duke IRB-Pro00069413) approved the parent project with a waiver of informed consent for nursing home residents.

### Development of the Nursing Home Severity Index–Pressure Injury

The NHSI-PrI is a measure of clinical severity for nursing home residents focused on the outcome of PrI risk and development. The first step in creating the NHSI-PrI was selecting the resident attributes to include those that are relevant to a nursing home population. The NHSI-PrI differs from prior clinical severity measures for acute, ambulatory, or rehabilitation care as it needs to account for greater variability in the length of time residents live in nursing homes and lower frequency of assessments, laboratory, and radiological tests. Skilled care in nursing homes often involves extended stays and uses different frequencies of diagnostics and treatments (including palliative care) from those of other care environments. Acute illness is less common, so laboratory tests are drawn infrequently and often only when there is an acute event. Assessments are made periodically such as at initial admission, quarterly, annually, and on condition change. Residents may need short- or long-term skilled care while recovering from an illness or surgery. Skilled care is characterized by wound and postsurgical care; injected medications; intravenous therapy; physical, occupational, and speech therapy; and regular monitoring of vital signs or disease-specific parameters such as blood glucose levels. Thus, a refined approach to severity measurement was needed to develop a meaningful profile of nursing home residents’ clinical severity.

International Classification of Diseases, Ninth Revision (ICD-9) and International Statistical Classification of Diseases, Tenth Revision (ICD-10) diagnosis codes used for residents within the study period were extracted and similar diagnoses combined (eg, codes for various types of pneumonia were aggregated into one severity criteria set including ICD-9 codes 055.1, 112.4, 136.3, 306.1, 480-486, 506.3, 507-507.1, 516.8, 517-517.1, 518.3, 668-668.04, 997.3, and 998.81, and ICD-10 codes J09.0-J18.9). For each diagnosis aggregate, a comprehensive set of relevant clinical severity indicators of resident attributes was derived from a combination of CSI criteria sets and other sources including Minimum Data Set (MDS) elements, nursing point of care documentation, and Risk Management System data elements: demographics (age, gender, race, and ethnicity) and clinical attributes (eg, laboratory test values; BMI categories, calculated as weight in kgs divided by height in m2: <18.5, 18.5-25.0, 25.1-30.0, 30.1-40.0, >40.0; weights/vital signs/pain data; and additional severity indicators of continence, dementia, locomotion, and dehydration). The inclusion of several of the NHSI severity indicators derived from the federally mandated assessment documentation for Medicare and Medicaid–certified long-term care facilities (MDS) used standardized clinical measures of functional capabilities and health needs specific to nursing home residents.

The second step in the NHSI-PrI development examined the associations and correlations of the severity indicators. Multiple indicators considered as alternative ways to describe the same resident attribute were combined into a single equivalence set (eg, highest or lowest pulse rate, electrocardiogram rhythm, and highest or lowest systolic and diastolic blood pressure to describe cardiovascular abnormality).

The third step developed algorithms to score the NHSI-PrI. A matrix was created to establish up to 4 severity levels for each indicator, their metrics, and the range of metric values applicable for nursing home residents: level 1 (normal to mildly abnormal), level 2 (moderate, nonsustained derangements that are not worrisome), level 3 (severe and worrisome derangements), and level 4 (most severe, catastrophic, life-threatening, or likely to result in organ failure). Equivalence sets were scored only once using the most abnormal indicator level during a specified time window to eliminate double scoring. Also, the most severe score of one or more indicator observations during a specified time window was used only once (eg, most abnormal body temperature recorded on different dates). The choice of severity levels was based on unsupervised methods using expert clinical judgment, literature, and clinical textbooks [10]. An expert panel of nurses and physicians on our research team reviewed the selected indicators and the associated 4 levels of severity thresholds necessary to create a measure of severity appropriate for nursing home residents. Based on previous literature and expert panel opinion, indicators were grouped into dimensions, laboratory, weights/vitals/pain, locomotion, and underweight. Next, expert panel reviewers interpreted the four indicator severity level scores as nonlinear and applied an exponential weighting function using a complex heuristic to create continuous NHSI-PrI dimension scores.

The final step in NHSI-PrI development involved validity testing. Secondary data from nursing home EHR, MDS, and Risk Management System data were used to validate the NHSI-PrI. The most commonly used measure in the United States to predict PrI risk, the Braden score [20], was examined in predictive models with and without severity dimensions. The Braden score is comprised of six subscales (sensory perception, mobility, activity, moisture, nutrition, and friction and shear) that are summed in a rating scale to help clinicians identify those at-risk for PrI development and to guide preventive measures based on risk factors. The subscales are rated from 1 to 4 (except friction and shear rated from 1 to 3), with 6-23 total points possible. Predictive validity varies by setting [21,22]. Risk categories for PrIs are based on total Braden scores: low (19-23), mild (15-18), moderate (13-14), and high (10-12) PrI risk.

A unique feature of the NHSI-PrI development used automatic severity scoring based on EHR, MDS, and Risk Management System data avoiding manual time-consuming abstraction. A computer algorithm was designed to generate the 4 severity levels according to extent of abnormality: the more abnormal the resident attributes, the higher the severity indicator levels and the NHSI-PrI’s severity dimension scores.

### Data and Data Management

Categories of EHR data used were vital signs, MDS elements, laboratory test values, and nursing point-of-care activities of daily living documentation. Data were extracted directly from EHRs with computer algorithms (code) created with SAS version 9.4 (SAS Institute Inc) [23]. All electronic data downloads were performed by the nursing home company in a Health Insurance Portability and Accountability Act–compliant format with the creation of a study identification number for each resident prior to data downloading and being transferred to Duke University’s secure drive space designated for the TEAM-UP study.

### Issues Defining Time Window of Exposure and Clinical Severity Measurements

Nursing homes conduct laboratory tests and other assessments infrequently, and enough time is needed to have sufficient data when resident clinical severity is most likely related to the outcome of interest. Also, residents’ severity measure comparisons depend on the standardization of an exposure window for the amount of time a person is observed and at risk for the outcome of interest. An exposure time window should reflect the period during which its effects are relevant to the specified outcome. Important factors to consider when defining exposure are the length of time, changes in exposure status, and consistency and accuracy of exposure measurements. Frequency, format, and intensity of residents’ observations are other important considerations. Clinical judgment was used to establish a 92-day window prior to the first PrI, which was a similar period to the typical quarterly Braden score and other resident assessments. For residents who did not develop PrIs, severity scores were based on indicator values during the final 92 days before discharge (death, transfer) from the nursing home or the end of the study period since residents are often sickest when they are older.

### Statistical Analysis

Descriptive statistics (means, SDs, frequencies, percentages) were used to describe demographic and clinical resident attributes with/without PrIs and were compared using 2-tailed *t* tests or chi-square tests as appropriate. A resident’s most severe Braden score (Worst-Braden) occurring during the 92 days prior to PrI development, discharge/death, or end of the study period was used to define risk categories of low (19-23), mild (15-18), moderate (13-14), and high (10-12). Validation methods included correlations overall and by Worst-Braden risk category followed by logistic regression models with/without severity dimensions to generate propensity scores or probabilistic estimates that a resident develops a PrI. Predictors (independent variables) included NHSI-PrI dimension scores and Worst-Braden score. The goal of these analyses was to assess the predictive capacity of three models: model 1 based on Worst-Braden scores alone, model 2 based on four NHSI-PrI dimensions, and model 3 based on Worst-Braden scores plus four severity dimensions.

Goodness of fit for logistic regression models was assessed in several ways. First, the overall models (relationship between the independent variables and the dependent variable) were assessed using a C statistic with a minimum value of 0.50 corresponding to chance and a maximum value of 1.0 (perfect prediction). To test the significance of improvement in fit after adding severity dimensions to the model, differences in C statistics between models with/without severity were examined using a likelihood ratio chi-square test. Second, the significance of each severity dimension was assessed by examining odds ratios (ORs) to determine the relative amount by which the odds of the dependent variable increased (OR≥1.0) or decreased (OR<1.0) when the value of the corresponding dimension variable increased by 1 unit. Third, the predictive accuracy or discriminating ability of the models was evaluated using propensity stratification. Observations were divided into equally sized strata defined by deciles of their sorted propensity scores to examine which model best discriminated between residents with/without PrIs. As a final validation of the NHSI-PrI, the study sample was randomly divided into a 65% training data set and a 35% validation data set, and the same validation statistics specified above were computed for each data set.

## Results

There were 1015 residents in 9 nursing homes during the study period who met the study inclusion criteria and had comprehensive EHR data in the relevant 92-day window for risk of PrI development. Across all 9 nursing homes, between 2.3% (n=3) to 18.3% (n=31) of residents developed PrIs for a total of 8.8% (n=89) having PrIs during the study period.

Table 1 compares attributes of residents with/without PrIs. There were no significant differences in age, gender, or race/ethnicity. However, the length of stay during the study period was significantly longer, although only 16 days, or 4.9%, for residents who developed PrIs versus those who did not. Significantly fewer residents with BMI ≥30 (n=21, 6.6%) and significantly more residents with BMI <18.5 (n=15, 18.5%) developed PrIs. Residents who developed a PrI had significantly lower (more severe) Worst-Braden scores and a greater percentage of residents in higher risk categories. All 4 of the NHSI-PrI severity dimensions (laboratory, weights/vitals/pain, locomotion, and underweight) indicated significantly greater clinical severity during the 92-day period before residents developed a PrI compared to the 92-day period prior to discharge for residents who did not develop PrIs.

Textbox 1 describes examples of clinical severity indicators contained in each of the 4 NHSI-PrI dimensions. The most abnormal values for these indicators during the 92-day window were used to quantify the severity of each indicator.

Different severity dimensions were associated with PrI development in residents classified by each of the Worst-Braden risk categories (Table 2). The higher the severity dimension score the more likely a PrI was to develop. The locomotion and underweight dimensions were significantly associated with PrI development for residents in low- and mild-risk categories, while the laboratory and weights/vitals/pain dimensions were significantly associated with PrI development for residents in moderate- and high-risk categories.

The C statistics from three logistic regression models captured the magnitude of improvement associated with adding severity dimensions to predictive models starting with the Worst-Braden score alone (Table 3). Age, gender, and race/ethnicity were not significant in predicting PrIs. The Worst-Braden score alone (model 1) provided limited predictive accuracy (C=0.627); the C statistic was 0.725 or 15.6% better using all four NHSI-PrI severity dimensions (model 2); C increased a little further to 0.748 or 19.3% better when the Worst-Braden score was added to the four NHSI-PrI dimensions (model 3), which improved the goodness of fit (model 1 vs model 3) significantly (*P*<.001).

The magnitude of this improvement is best gauged by examining the ORs of the individual severity dimensions. For model 3, an increase of 5 points in the locomotion dimension score increases the likelihood of PrI by 75%. A 5-point increase in the underweight dimension score increases the likelihood of PrI by 50%.

**Table 1:** Comparison of characteristics for residents without and with pressure injuries (PrIs) during the preintervention time period (N=1015).

Demographic and clinical characteristics		Total population (N=1015)	Residents without PrI (n=926)	Residents with PrI (n=89)	*T* test, *F* test, or chi-square (*df*)	*P* value
Resident age (years), mean (SD)		77.94 (12.9)	77.85 (12.9)	78.87 (12.5)	–0.71 (1013)	.48
Male, n (%)		357 (35.17)	329 (35.5)	28 (31.5)	0.77 (1013)	.44
**BMI (kg/m^2^), n (%)**					18.33 (3)	<.001
	<18.5	81 (8.0)	66 (7.1)	15 (16.9)		
	18.5 to <25	383 (37.7)	341 (36.8)	42 (47.2)		
	25.0 to <30	233 (23.0)	222 (24.0)	11 (12.4)		
	>30	318 (31.3)	297 (32.1)	21 (23.6)		
**Race/ethnicity, n (%)**					1.36 (2)	.51
	Asian	32 (3.2)	29 (3.1)	3 (3.4)		
	Black	333 (32.8)	299 (32.3)	34 (38.2)		
	White	650 (64.0)	598 (64.6)	52 (58.4)		
Braden-First score^a^, mean (SD)		18.00 (2.9)	18.13 (2.9)	16.66 (2.7)	4.57 (108.80)	<.001
Braden-MEAN score^b^, mean (SD)		17.61 (2.7)	17.78 (2.7)	15.84 (2.2)	7.75 (115.41)	<.001
Braden-Worst score^c^, mean (SD)		16.43 (3.3)	16.55 (3.3)	15.19 (2.7)	4.42 (115.11)	<.001
**Braden-MEAN risk categories based on Braden-MEAN scores^b^, n (%)**					20.95 (3)	<.001
	Low risk (score 19-23)	469 (46.2)	448 (48.4)	21 (23.6)		
	Mild risk (score 15-18)	434 (42.8)	382 (41.3)	52 (58.4)		
	Moderate risk (score 13-14)	82 (8.1)	71 (7.7)	11 (12.4)		
	High risk (score 10-12)	30 (3.0)	25 (2.7)	5 (5.6)		
**Braden-Worst risk categories based on Braden-Worst scores^c^, n (%)**					12.17 (3)	.007
	Low risk (score 19-23)	282 (27.8)	270 (29.2)	12 (13.5)		
	Mild risk (score 15-18)	442 (43.6)	401 (43.3)	41 (46.1)		
	Moderate risk (score 13-14)	171 (16.9)	149 (16.1)	22 (24.7)		
	High risk (score 10-12)	120 (11.8)	106 (11.5)	14 (15.7)		
Weights/vitals/pain severity dimension score, mean (SD)		14.70 (12.2)	14.37 (12.2)	18.16 (11.8)	–2.81 (1013)	.005
Locomotion (On_Off) severity dimension score, mean (SD)		2.34 (3.1)	2.20 (3.0)	3.83 (3.7)	–4.08 (99.37)	<.001
Laboratory severity dimension score, mean (SD)		6.22 (11.1)	5.71 (10.5)	11.54 (15.1)	–3.56 (96.44)	<.001
Length of stay-total^d^ (days), mean (SD)		1349 (1287)	1326 (1287)	1591 (1270)	–1.86 (106.15)	.06
Length of stay during preintervention study period^e^ (days), mean (SD)		327 (821)	326 (83)	342 (66)	–2.11 (116.37)	.04

^a^Braden-First score: first Braden score occurring during the preintervention period.

^b^Braden-MEAN score: mean of all Braden scores occurring during the preintervention period.

^c^Braden-Worst score: worst Braden score occurring during the 92 days prior to pressure injury development, discharge/death, or end of the preintervention period.

^d^Length of stay total: number of days from nursing home admission to end of preintervention period, mean (SD).

^e^Length of stay preintervention study period: number of days during preintervention time period, mean (SD).

**Description of the Nursing Home Severity Index-Pressure Injury (NHSI-PrI) clinical severity dimensions and their indicators. Weights for each indicator comprising a dimension are summed to produce a dimension score.**

**Laboratory dimension**

- Lowest platelets (10^3^/uL), lowest female hemoglobin (HGB; g/dl), lowest female hematocrit (HCT; %), lowest male HGB (g/dl), lowest male HCT (%)
- Highest glucose (mg/dl), highest hemoglobin A_1c_ (n x norm)
- Highest/lowest potassium (K; mEq/L)
- Highest blood urea nitrogen (mg/dl), highest creatinine (mg/dl), lowest albumin (mg/dl)
- Highest aspartate transaminase (serum glutamic-oxaloacetic transaminase; n x norm), highest alanine transaminase (serum glutamic-pyruvic transaminase; n x norm)
- Highest sodium (NA; mEq/L), lowest sodium (mEq/L)
- Highest 24 hr urine protein (mg/dl), highest urine protein via dipstick
- O_2_ saturation on pulse oximetry (%), arterial blood gases, lowest pH (no units), lowest pO_2_ (mm/Hg), highest pH (no units), lowest total venous CO_2_ (mEq/L)
- Highest white blood cell count (WBC; k/cu mm), highest bands (%), lowest WBC (k/cu mm)
- Lowest lymphocytes (%)
- Highest total bilirubin (mg/dl)
- Highest total calcium (mg/dl)
- Highest alkaline phosphatase (u/l)

**Underweight dimension**

- BMI <18.5 kg/m^2^

**Weights, vitals, pain dimension**

- Infiltrates/consolidation in lungs, rales/rhonchi/wheezes, dyspnea, breath sounds, kussmaul breathing, sputum/secretions
- Highest temperature, rigors/chills, lowest temperature
- Highest pulse rate, electrocardiogram rhythm, highest blood pressure systolic, highest blood pressure diastolic, lowest pulse rate, lowest systolic blood pressure, orthostatic blood pressure
- Weight loss, cachexia, weight gain, general pain
- Pulse characteristics
- Chest pain

**Locomotion dimension (locomotion dimension indicators are calculated as average/day frequency)**

- Locomotion OFF unit with wheelchair
- Locomotion OFF unit with wheeled recliner
- Locomotion OFF unit one person assist
- Locomotion OFF unit total dependence
- Locomotion ON unit one person assist
- Locomotion ON unit total dependence

**Table 2:** Correlations among predictor variables and outcome of pressure injury used in logistic regression models.

Predictor variable		Total residents (N=1015; PrI^a^ n=89)	Pressure injury low risk (n=282; PrI n=12)	Pressure injury mild risk (n=442; PrI n=41)	Pressure injury moderate risk (n=171; PrI n=22)	Pressure injury high risk (n=120; PrI n=14)
**Worst-Braden**						
	*r*	–0.12	–0.09	–0.12	0.02	0.13
	*P* value	<.001	.12	.01	.79	.17
**Laboratory dimension**						
	*r*	0.15	0.07	0.12	0.22	0.29
	*P* value	<.001	.22	.01	.003	.001
**Weights/vitals/pain dimension**						
	*r*	0.09	0.28	0.09	0.09	0.19
	*P* value	.004	.64	.05	.23	.04
**Locomotion dimension**						
	*r*	0.15	0.21	0.15	0.09	0.10
	*P* value	<.001	<.001	.002	.23	.26
**Underweight dimension**						
	*r*	0.10	0.17	0.11	0.08	0.02
	*P* value	.001	.005	.02	.32	.83

^a^PrI: pressure injury.

**Table 3:** Logistic regression models predicting pressure injury development.

Logistic regression model		Estimates	Standard error	*P* value	Odds ratio (95% CI)	C statistic (*df*)	Likelihood ratio chi-square test	
							Chi-square (*df*)	*P* value
**Model 1 (Braden)**						0.627 (1)	N/A^a^	N/A
	Worst-Braden score	–0.126	0.035	<.001	0.88 (0.82-0.94)			
**Model 2 (NHSI-PrI^b^)**						0.725 (4)	50.33 (4)^c^	<.001
	Underweight dimension	0.098	0.036	.006	1.10 (1.03-1.18)			
	Laboratory dimension	0.030	0.008	<.001	1.03 (1.01-1.05)			
	Weights/vitals/pain dimension	0.024	0.009	.009	1.02 (1.01-1.04)			
	Locomotion dimension	0.149	0.034	<.001	1.16 (1.09-1.24)			
**Model 3 (Braden + NHSI-PrI)**						0.748 (5)	61.72 (5)^d^	<.001
	Worst-Braden score	-0.121	0.037	.001	0.89 (0.82-0.95)			
	Underweight dimension	0.097	0.360	.007	1.10 (1.03-1.18)			
	Laboratory dimension	0.031	0.008	<.001	1.03 (1.02-1.05)			
	Weights/vitals/pain dimension	0.025	0.009	.007	1.03 (1.01-1.04)			
	Locomotion dimension	0.140	0.034	<.001	1.15 (1.08-1.23)			

^a^N/A: not applicable.

^b^NHSI-PRI: Nursing Home Severity Index–Pressure Injury.

^c^Comparing goodness of fit of two models: model 2 versus model 1.

^d^Comparing goodness of fit of two models: model 3 versus model 1.

**Figure 1:**
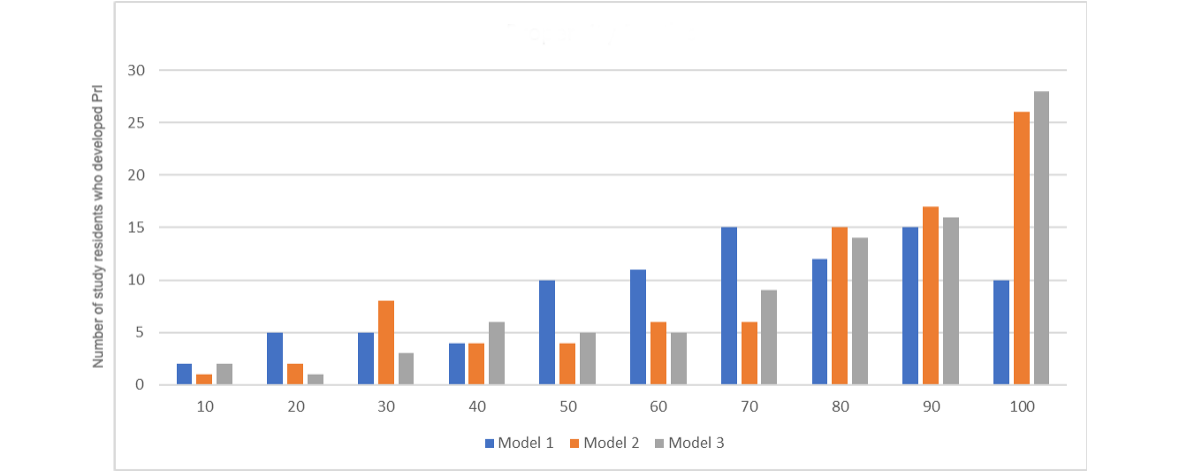
Propensity deciles for all models. PrI: pressure injuries.

The histogram in Figure 1 summarizes the propensity score results generated from prediction models using model 1 (only Worst-Braden score) versus model 2 (only 4 NHSI-PrI dimensions) or model 3 (Worst-Braden score plus 4 NHSI-PrI dimensions). The top decile for each model contains 10% of the population most likely to develop a PrI and the bottom decile contains 10% of the population with the lowest likelihood of PrI. The deciles and number of study residents who actually developed PrIs in that decile are graphed on the x and y axes, respectively. Models 2 and 3 exhibit patterns of mostly “staircase” increases for each decile demonstrating that the models “binned” the residents correctly from those least likely to develop a PrI to most likely. In contrast, model 1 exhibits an irregular pattern for each decile, both up and down, indicating that the model is not doing as good a job of predicting a resident’s likelihood for PrI. More than 65% (n=58) of residents with PrIs are identified in the three highest propensity deciles using models 2 and 3 compared to only about 42% (n=37) of residents with PrIs in the three highest deciles using model 1. Thus, using propensity score analysis, the inclusion of severity dimensions in models 2 and 3 resulted in the identification of 21 more residents at greater risk (in the three highest propensity deciles) of developing a PrI than in model 1.

The outcome of PrI development was also used to validate the NHSI-PrI results for training and validation data sets. The randomly selected training data set contained 56 PrIs in 658 residents, and the validation data set contained 33 PrIs in 357 residents. For training data, the corresponding predicted C statistics were 0.618 (model 1), 0.717 (model 2), and 0.735 (model 3), resulting in an 18.9% improvement from model 1 to 3. For validation data, the C statistics were 0.648 (model 1), 0.810 (model 2), and 0.816 (model 3), resulting in a 25.9% improvement from model 1 to 3.

## Discussion

### Principal Findings

Multiple different approaches were used to validate the NHSI-PrI to predict residents at risk for PrI development. Model statistics improved from using the Worst-Braden score alone (C=0.627) to using NHSI-PrI alone (C=0.725) to combining the Worst-Braden score and NHSI-PrI (C=0.748). Looking at propensity score deciles versus actual results also validated the improvement indicated by NHSI-PrI. Finally, randomly dividing the data into training and validation data sets showed that the training values had similar corresponding C statistics for Worst-Braden scores alone versus NHSI-PrI alone versus the combination of the two.

Measuring resident clinical severity and predicting a specific outcome such as PrI involves an examination of numerous resident attributes (eg, physiologic, functional, and psychosocial variables during a specified window of time) and potentially hundreds of data points. Using existing and relevant data, nursing home outcomes can only be evaluated accurately when pertinent resident attributes that impact the resulting outcomes are included. There is no way to demonstrate whether differences in outcomes are associated with either health interventions, differences in clinical severity, or both if critical aspects of a resident’s clinical severity are not included.

Clinical severity in nursing home residents is challenging to define given the multitude of factors affecting the overall health status of older adults who are potentially further compromised by residing in a nursing home [2]. Significant differences are evident in clinical severity definitions for adults in differing care settings. For example, a severity indicator label may be the same for an adult cared for in acute care or a nursing home setting, but nursing home resident outcome prediction required modification in that indicator’s thresholds due to substantial differences in age-related attributes. Identification of new severity indicators and new thresholds for some of those indicators were needed when applied to older adult residents.

Multiple different data sources with varied recording formats and coding patterns for the same indicator were encountered in developing and programming the new NHSI-PrI measure, making synchronization of data elements challenging. Yet, it was required to avoid subsequent issues interpreting analysis results.

### Strengths

The new NHSI-PrI measure has two unique features: (1) capacity for automatic scoring and (2) daily calculation. First, the NHSI-PrI was designed by clinical experts to be scored automatically from downloaded structured EHR data including vital signs, MDS data elements, laboratory test values, weights, etc. Second, the NHSI-PrI measure can be calculated daily based on findings during the most recent prior 92-day time window, allowing for evolving clinical severity changes to be monitored over time.

There is a substantial benefit to identifying and monitoring known PrI predictors and improving prediction using electronic data in addition to the existing Braden score. Little is known about differences in who does and does not develop a PrI, especially among nursing home residents. PrI prevention efforts are well established according to international guidelines, yet PrI incidence has remained high in nursing homes. Historically, clinically assessed PrI risk among residents has resulted in most preventive resources being allocated to residents evaluated at moderate or high risk. However, significant numbers of PrIs also occur in residents in low and mild Worst-Braden risk categories [5]. This research supports the value of a well-discriminating model that differentiates residents with a higher likelihood of developing a PrI from those with a lower likelihood. These findings suggest that relying on the Worst-Braden score alone is a weak predictor. The use of the Worst-Braden score in combination with four severity dimensions of the NHSI-PrI significantly enhances the accuracy of PrI prediction. This new knowledge can be used to design and modify resident-specific PrI prevention plans. Thus, the addition of specific NHSI-PrI dimensions to current risk assessment resources has the potential to substantively impact quality care decisions aimed at improving PrI prevention outcomes, especially among different Worst-Braden risk categories.

### Limitations

This study had several limitations that may affect the reproducibility and generalizability of results. First, nursing home populations have several unique characteristics that provide challenges for identifying comparable discrete times of exposure or defined time frames. For example, it was assumed that residents’ exposure time had a clearly defined start and end date when in fact this varied across residents. An up to 92-day window was determined to be most clinically relevant and applied to define exposure duration to measure and compare clinical severity for residents with and without PrIs. Results may differ if shorter or longer time windows are applied. The approach used in computing the NHSI-PrI directly from downloaded structured electronically available data may have limited the variety of indicators that could be included in the NHSI-PrI and may need updating as more relevant structured electronic data become available.

Second, our models are not directly linked in real time to measures for risk mitigation. This is due to the fact that clinical severity data in nursing homes are captured less frequently than ongoing clinical appraisals in other settings. Some observations that may be useful predictors are recorded only every quarter. This limits the precision of risk indicators that can be used. More frequent measurement of relevant severity clinical indicators would likely improve the predictive ability of NHSI-PrI.

### Future Directions

Substantive strides are needed to standardize health care data to facilitate process improvements in data interpretation for future studies. Determination of severity levels required complex data interpretation from various sources for which there is currently no data field standardization. The substantial amount of coding across electronic data formats was a fundamental challenge. Data values needed to be converted to equivalents and interpreted for descriptive data fields to assign severity levels. Data standardization and interpretive processes were carefully performed, checked, and further evaluated taking clinical judgment into account. This process required significant effort to minimize inconsistencies.

Finally, the effectiveness of the NHSI-PrI using a larger sample of nursing home facilities and residents is unclear. Our sample was divided into training and validation data sets, each of which well represented the whole data set to test generalizability. Larger confirmatory studies with a different cohort of nursing home residents and facilities should establish the reliability and validity of the new NHSI-PrI and its results.

### Conclusions

The newly created NHSI-PrI was successful in developing a meaningful profile of clinical severity among nursing home residents and accurately predicting the risk of PrI development. Findings support that clinical severity dimension scores can be used in combination with Worst-Braden scores to augment PrI prediction and potentially impact the quality of care decisions aimed at improving individual PrI prevention plans.

## Abbreviations

CSI: Comprehensive Severity Index
EHR: electronic health record
ICD-9: International Classification of Diseases, Ninth Revision
ICD-10: International Statistical Classification of Diseases, Tenth Revision
MDS: Minimum Data Set
NHSI: Nursing Home Severity Index
NHSI-PRI: Nursing Home Severity Index–Pressure Injury
OR: odds ratio
PrI: pressure injury
TEAM-UP: Turn Everyone and Move for Ulcer Prevention

## Data Availability

The data used in this publication include protected health information and therefore cannot be freely shared. Data sharing will be possible with case-by-case approval from the authors’ institutional review board; requests may be directed to the principal investigator.
